# Fluoride and children’s IQ: evidence of causation lacking

**DOI:** 10.1038/s41432-024-01022-6

**Published:** 2024-06-01

**Authors:** Deborah Moore, Anne-Marie Glenny

**Affiliations:** 1 Senior Clinical Lecturer / Honorary Consultant in Dental Public Health, The University of Liverpool, Liverpool, UK; 2 Professor of Health Sciences Research, The University of Manchester, Manchester, UK

**Keywords:** Fluoridation, Dentistry

## Abstract

**Data sources:**

Human, animal, and in vitro studies. Extensive literature search of multiple bibliographic databases, trial registries, major grey literature sources and bibliographies of identified studies.

**Study selection:**

The authors aimed to identify studies which could be used to determine the maximum safe level for fluoride in drinking water. To identify new studies published since a 2016 Australian review, the search period was 2016 to July 2021. Studies which evaluated the association between either naturally or artificially fluoridated water (any concentration) and any health outcomes were included. No restrictions on study design or publication status. Articles published in a ‘non-Latin language’ were excluded. Screening of abstracts and full texts was in duplicate. For IQ and dental fluorosis, a top-up search was conducted between 2021 and Feb 2023.

**Data extraction and synthesis:**

Extensive data extraction. Risk of bias assessment using the OHAT tool. A narrative synthesis of the results was carried out.

**Results:**

The review included 89 studies in humans, 199 in animals and 10 reviews of *in vitro* studies. Where there was consistent evidence of a positive association, in relation to a water fluoride concentration of <20 ppm (mg F/L), and where studies were judged to be acceptable or high quality, health effects were taken forwards for further examination of causality using Bradford Hill’s 9 criteria. Of the 39 health outcomes reviewed, 4 were further assessed for causality. The authors reported ‘strong’ evidence of causality for dental fluorosis and reductions in children’s IQ scores, ‘moderate’ strength evidence for thyroid dysfunction, ‘weak’ for kidney dysfunction, and ‘limited’ evidence for sex hormone disruption.

**Conclusions:**

The authors conclude that moderate dental fluorosis and reductions in children’s IQ scores are the most appropriate health outcomes to use when setting an upper safe level of fluoride in drinking water. For reductions in children’s IQ, the authors acknowledge a biological mechanism of action has not been elucidated, and the dose response curve is not clear at lower concentrations, limiting the ability to set an upper safe threshold.

## A Commentary on

**Taher M K, Momoli F, Go J et al**.

Systematic review of epidemiological and toxicological evidence on health effects of fluoride in drinking water. *Crit Rev Toxicol* 2024 **54** 2–34.

**GRADE Rating**: 

## Commentary

The safety of water fluoridation has been frequently questioned and concerns have been raised about many different health effects. Recently, studies investigating neurodevelopment and IQ have generated increasing attention. In the USA, there is an ongoing court case *‘Food & Water Watch, Inc.* et al. *v. Environmental Protection Agency (EPA)* et al.’ in which the plaintiffs, a coalition of anti-fluoridation groups, claim that fluoride poses an ‘unreasonable risk’ to health on the grounds that it is neurotoxic, and should be regulated as such under the Toxic Substances Control Act of 1976 (TSCA)^1^.

A key piece of evidence in the trial is a US National Toxicology Programme (NTP) systematic review of fluoride exposure and neurodevelopmental and cognitive health effects. The NTP systematic review was started in 2016 and the first draft (2019) concluded that “fluoride is presumed to be a cognitive neurodevelopmental hazard to humans”. The US National Academies of Science, Engineering and Medicine (NASEM) have twice peer reviewed the draft NTP review and requested revisions on the basis that the conclusions were not adequately supported by the evidence^2^.

An important issue is that many of the studies demonstrating adverse effects of fluoride on IQ are related to very high naturally occurring water fluoride concentrations, far higher than the recommended (and legal, in the UK and Europe) maximum of 1.5 mg F/L, and the 1 mg F/L target for water fluoridation programmes. Additionally, many studies have been conducted in developing countries in populations who may also be exposed to environmental pollutants such as lead, mercury or arsenic through contaminated drinking water or coal smoke pollution^2,3^. At the time of writing, the final NTP review has not been published, however, the most recently available third draft (Sept 2022) is more cautious, stating with “moderate confidence” that “higher fluoride exposure… [>1.5 mg F/L] is consistently associated with lower IQ in children”, and acknowledges that “more studies are needed to fully understand the potential for lower fluoride exposure [<1.5 mg F/L] to affect children’s IQ”^4^.

This brings us back to the present systematic review, by Taher et al.^5^. In terms of the methods, the scope of the review is extensive. The main manuscript is 29 pages, and there are over 1000 pages of supplementary materials. It is therefore surprising that there is no indication that the review protocol was registered in advance, important to maintain rigour and reduce bias. Relatedly, there is no justification given for the ‘top-up search’ between 2021 and Feb 2023, for only IQ-effects studies. It is not clear that data extraction was performed in duplicate, or that the data extraction forms were piloted. Indeed, a large volume of information has been extracted but not all of it is used in the analysis or manuscript, and there are some reporting inconsistencies.

Our main area of concern reflects the risk of bias assessment and how it has been applied. The authors used the OHAT Tool for Human and Animal Studies. A requirement of the tool is that important confounding factors should be agreed in advance with subject matter experts, for each health outcome. There is no indication that this was done, therefore we have limited confidence in the authors’ assessment as we are not sure what confounders they would expect to be considered as a minimum. In addition, the study summary table in the main manuscript presents study type, country, and direction of association for each health outcome, but does not include any indication of the strength of the association or the water fluoride concentration, which makes it difficult to evaluate the relevance of the findings to water fluoridation programmes.

The human evidence included in the Taher review overlaps with the draft NTP review^4^, and other systematic reviews on the neurological and health effects of fluoride in water, including a recent Irish government review (Lambe et al.^6^), and a Canadian government review (CADTH)^7^. There appear to be differing perspectives on the quality of this evidence from reviewers with a toxicology perspective (Taher et al.^5^ and the draft NTP review), versus those with an evidence-based medicine perspective^6,7^. The latter have stated that the current evidence base related to IQ is insufficient to draw conclusions, and that further high quality research is needed (Lambe et al. and CADTH)^6,7^.

To illustrate, a Canadian prospective cohort study by Till. et al.^8^, assessed as ‘high quality / low risk of bias’ by both Taher et al. and the draft NTP review^4^, was assessed as ‘low quality / high risk of bias’ by the Lambe et al. and CADTH reviews^6,7^. Bias concerns stated were: selection bias (low participation rate, high loss to follow-up); validity of the fluoride and IQ measurements; and insufficient adjustment for confounding, including by maternal IQ and marital status^6,7^. Similar differences in risk of bias assessments are evident for other papers included in the multiple reviews. The paper by Till et al. (2020) uses data from the Maternal-Infant Research on Environmental Chemicals (MIREC) birth cohort database in Canada, as do several other recent fluoride-IQ papers (including Farmus et al.^9^, also assessed as high quality by Taher et al., and low quality by Lambe et al.^6^). The MIREC cohort was not designed to evaluate fluoride and the studies using these data have been extensively critiqued^2,10^, with recent authors stating that these studies should be “considered unacceptable for legal and policy purposes”^11^.

In conclusion, there is ongoing and high-profile debate regarding the impact of fluoride in drinking water on IQ, with very different perspectives on bias according to discipline. High-quality prospective longitudinal studies based on individual-level exposures, in populations exposed to fluoride concentrations of relevance to fluoridation programmes, and taking account of all important confounding factors are necessary to provide higher-quality information.

Practice Points

- Practitioners may need to respond to patient concerns regarding the neurodevelopmental toxicity of fluoride in drinking water.
- Recent proposals to extend water fluoridation in the North East of England mean this issue is very topical.
- Practitioners should be aware the evidence base is much disputed with ongoing concerns regarding the validity, applicability, and risk of bias in many of the studies.
